# High-intensity interval training reduces inflammatory mediator levels in the testes of spontaneously hypertensive rats

**DOI:** 10.1590/1984-3143-AR2024-0024

**Published:** 2025-05-09

**Authors:** Ronivania Jenuario Silva Nespolo, Allice Santos Cruz Veras, Lauren Chrys Soato Marin, Margarete Jardinetti de Oliveira, Aline de Oliveira Santos, Evellin Heloisa Paulineli Pereira, Francilene Lima Agostinho de Souza, Francis Lopes Pacagnelli, Caliê Castilho, Giovana Rampazzo Teixeira, Robson Chacon Castoldi, Ines Cristina Giometti

**Affiliations:** 1 Universidade do Oeste Paulista, Presidente Prudente, SP, Brasil; 2 Programa de Pós-graduação Multicêntrico em Ciências Fisiológicas, Sociedade Brasileira de Fisiologia, Universidade Estadual Paulista, Presidente Prudente, SP, Brasil; 3 Faculdade de Ciências e Tecnologia, Universidade Estadual Paulista, Presidente Prudente, SP, Brasil; 4 Programa de Pós-graduação em Exercício Físico na Promoção da Saúde, Universidade do Norte do Paraná, Londrina, PR, Brasil

**Keywords:** exercise, hormonal dosage, interleukin, testosterone, TNFα

## Abstract

Hypertension is an age-related pathology that causes a decline in the function of all organ systems, including the reproductive system, due to its association with increased oxidative stress and inflammation. The inflammatory cytokine levels increase as a result of hypertension and cause inflammation and tissue injury. Although high-intensity interval training (HIIT) has shown promise as a nondrug treatment for hypertensive individuals, its effects on the reproductive system of hypertensive individuals remain unknown. The aim of this study was to investigate the effects of HIIT on plasma hormone concentrations and the expression of inflammatory mediators in the testes of spontaneously hypertensive rats (SHRs). Male SHRs were divided into 2 groups: SHR (control, n=8) and HIIT (SHRs subjected to HIIT on a treadmill for 8 weeks, n=9) groups. The expression of inflammatory mediators (TNFα and IL-6) in the testes and testosterone, prolactin, and corticosterone concentrations in plasma were measured. No difference in TNFα expression was found between the groups. The groups also showed no significant differences in hormone levels. However, SHRs that underwent HIIT showed lower immunostaining for IL-6 in their testes than did SHRs that did not undergo HIIT training (P < 0.05) and the HIIT group presented lower lower systolic blood pressure than did the SHR group. We concluded that HIIT for two months reduces the BSP and IL-6 levels in the testes of hypertensive rats.

## Introduction

Arterial hypertension is the leading cause of cardiovascular disease and results in the death of 10 million people per year (Frieden and Jaffe, 2018) and the annual medical cost of hypertension is estimated at $370 billion worldwide (Saco‐Ledo et al., 2020). More than two-thirds of the world’s population over 65 years of age has hypertension (Sun, 2015).

Hypertension causes oxidative stress and inflammation, which together are responsible for vascular damage, including endothelial dysfunction, vascular hypertrophy, and fibrosis (Didion, 2017; Guzik and Touyz, 2017; Handy and Loscalzo, 2017). Inflammatory mediators of hypertension, such as tumor necrosis factor alpha (TNFα), stimulate the production of reactive oxygen species (ROS) and thereby increase hypoxia-inducing factor (HIF1α), which causes tissue hypoxia (Handy and Loscalzo, 2017). Inflammatory cytokines (interferons, interleukins, and TNFα) lead to the infiltration of T cells and macrophages, which contributes to tissue injury (Guzik and Touyz, 2017). Interleukin 6 (IL-6) increases as a result of hypertension and has important effects on blood vessels, including endothelial activation, immune cell recruitment, vascular permeability, vascular hypertrophy and fibrosis, and endothelial dysfunction, contributing to increased blood pressure (Didion, 2017).

Approximately 700 million men of reproductive age have hypertension (Mills et al., 2016). Hypertension can affect the reproductive organs of humans, causing decreases in the quality and volume of semen and in the motility and quantity of spermatozoids (Guo et al., 2017). Sperm quality is an indicator of male health because men with normal spermatogenesis have greater longevity (Colli et al., 2019). Erectile dysfunction in humans is positively correlated with hypertension (Foy et al., 2019). Hypertension is also correlated with lower total serum testosterone and free testosterone levels (Navaneethabalakrishnan et al., 2020). Hypertension in rats alters the morphology of the seminiferous tubules, decreases spermatogenesis (Altıntaş Aykan, 2020) and sperm concentration and motility, and increases abnormalities in sperm morphology (Akomolafe et al., 2022). Spontaneously hypertensive rats (SHRs) present vascular alterations that lead to disorders in the seminiferous tubules, with consequent depletion of germ cells, impaired spermatogenesis and lower erection reflexes (Azu, 2015).

The levels of several inflammatory cytokines, such as TNFα, IL-1β, IL-6, and IL-8, are elevated in the seminal plasma of men with impaired fertility, although the cellular immune response in relation to fertility has been poorly studied (Djourabchi Borojerdi et al., 2020). Leukocytopenia is associated with the production of ROS and cytokines (Djourabchi Borojerdi et al., 2020). Seminal plasma IL-6 is negatively correlated with the motility and viability of sperm in humans (Kopa et al., 2005). The IL-6 receptor (IL-6R) has been detected in the middle piece of human spermatozoa, and analyses have revealed positive correlations among its immunolocalization, the level of IL-6 in seminal plasma, and the sperm count, and a negative correlation between IL-6R and sperm motility (Djourabchi Borojerdi et al., 2020). An increase in macrophages and IL-6 in the testes can negatively influence spermatogenesis through disturbance of the blood barrier and infertility (Hussein et al., 2005). Furthermore, TNFα and IL-6 levels are increased in the seminal plasma of obese men, affecting sperm quality (Han et al., 2017). In another study, increases in IL-6 and TNFα were detected in men with varicocele (Micheli et al., 2019).

Studies have shown that physical exercise reduces the risk of cardiovascular incidents in hypertensive individuals (Li et al., 2017), and the effects on hypertension and mortality depend on the type of physical exercise (MacDonald et al., 2020; Oja et al., 2017). More vigorous physical activities, such as running, are associated with lower risks of mortality from hypertension (Wen et al., 2011). Some studies have revealed that high-intensity interval exercise (HIIT) can also be indicated for cardiac patients (Ciolac et al., 2011; Engel et al., 2022; Tjønna et al., 2008). In obese mice, both moderate- and high-intensity physical exercise decrease body fat, but only moderate exercise decreases oxidative stress and inflammation, which impair reproductive function and thus improve testosterone biosynthesis and sperm quality (Yi et al., 2020). Furthermore, HIIT decreases inflammatory cytokine levels in the prostate of hypertensive animals (Correia et al., 2022).

Therefore, the aim of this study was to verify the effects of HIIT on the expression of inflammatory mediators (TNFα and IL-6) in the testes and on hormonal concentrations in SHRs.

## Methods

### Experimental animals

SHRs aged 12 months at the beginning of the experiment were divided into 2 groups: the SHR group (SHRs that did not undergo HIIT, n=9) and the HIIT group (SHRs that underwent HIIT, n=10). The mean weights of the animals were 316.02 g (305.3-329.1 g) in the SHR group and 315.33 g (311.4-321.1 g) in the HIIT group. The animals were placed in plastic boxes measuring 41x34x16 cm, and 3-5 animals were included in each box. The rats were exposed to temperatures ranging from 21°C to 23°C, 50% to 60% relative humidity, and inverted light cycles consisting of 12 hours of light and 12 hours of dark. The animals received commercial balanced feed (Supralab® produced by Alisul, Rio Grande do Sul, Brazil) and water *ad libitum*.

All experimental protocols used were in accordance with the principles of the Care for Laboratory Animals formulated by the Brazilian College of Animal Experimentation (COBEA) and in accordance with the Guide for the Care and Use of Laboratory Animals (2011) published by the *National Research Council*. All procedures used were approved by the Research Ethics Committee of the Universidade do Oeste Paulista (UNOESTE), Presidente Prudente (SP), protocol number 4418.

### HIIT training

**Figure 1:**
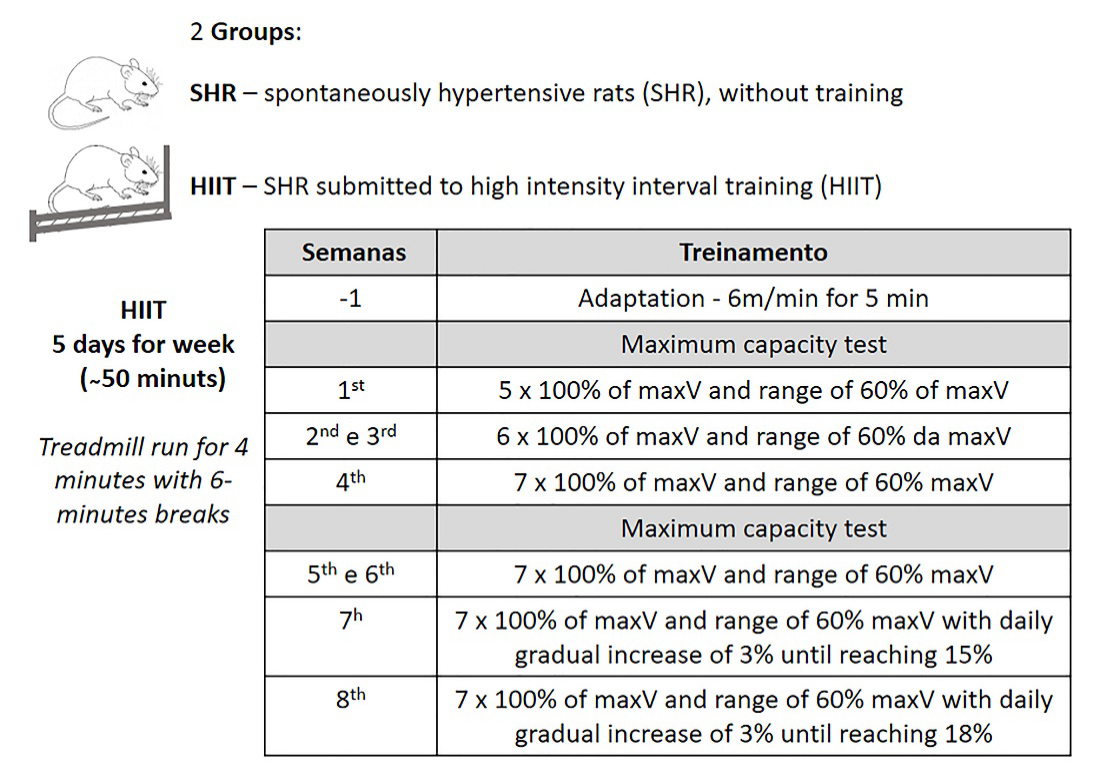
Experimental design of the group division and high-intensity interval training (HIIT) performed by the HIIT group. maxV = maximum exhaustion velocity, which was considered the velocity at which the rats refused to run even after stimulation or were unable to coordinate their steps.

After an adaptation period of one week, the training protocol was carried out on a treadmill adapted for rodents (model TK 1, IMBRAMED) (Pacagnelli et al., 2014) for five consecutive days each week over a period of 8 weeks; each session consisted of approximately 50 minutes (Fig. 1) (Correia et al., 2022; Moreira et al., 2013; Zamberlan Ferreira et al., 2017), and the maximum exhaustion velocity was considered. Rats were considered exhausted when they refused to run even after stimulation or when they were unable to coordinate their steps (Rossoni et al., 2011).

### Systolic blood pressure (SBP)

For the assessment of pressure control, SBP was measured two days before the experiment began and three days after the training was finished via plethysmography using the tail cuff method (Narco Bio-System®, model 709-0610, International Biomedical, Inc., USA). Each animal was individually coupled the system, and the average of two readings was recorded for each measurement. Before the measurements, the animals were maintained at 37°C for 15 minutes, to promote dilatation of the caudal artery.

### Testicular processing

Two days after the end of the HIIT programme, the animals were weighed, anaesthetized with ketamine hydrochloride (50 mg/kg, i.p.) and xylazine hydrochloride (10 mg/kg, i.p.) and euthanized by decapitation. Portions (~100 mg) of the right testes of the rats were collected, immersed in TRIzol® (Thermo Fisher Scientific®), immediately frozen in liquid nitrogen, and stored in a freezer at -80°C for analysis of the expression levels of the *Il6* and *Tnfα* genes. The remaining testes (left testis of each rat) were harvested, cleaned, and immersed in Methacarn fixative solution (60% methanol, 30% chloroform, and 10% acetoacetic acid). After 2 h, a small cut was made in the pole regions of each testis, and 22 hours later, the testes were washed, the pole regions were discarded, and the solution was replaced with 70% alcohol, where the testis samples remained until processing. The samples were subsequently embedded in paraffin to obtain 3 μm thick sections, which were subjected to immunohistochemistry for detection of the IL-6 and TNFα proteins.

### Gene expression

For RT-qPCR analysis, testis samples, stored in TRIzol® (Thermo Fisher Scientific®) in a freezer at -80°C, were ground in a tissue homogenizer and subjected to the TRIzol® extraction protocol. The concentration of total RNA recovered was measured by spectrophotometry. All total RNA samples were treated with DNAse before being subjected to reverse transcription (RT) followed by quantitative polymerase chain reaction (qPCR) according to the instructions of the DNAse I protocol - Amplification Grade (Invitrogen®).

RT was performed using the High-Capacity cDNA Reverse Transcription Kit (Applied Biosystems®) following the manufacturer’s protocol with random oligonucleotide primers.

qPCR was performed for the quantitative analysis of relative gene expression. As an internal control for the qPCRs, 3 reference genes were used to normalize the results obtained for the target genes. The oligonucleotide primers used were obtained from TaqMan® assays (Applied Biosystems®, Foster, USA) and were standardized as described below for the following target genes: *Tnfα* (Assay Rn01525859_g1, GenBank NM_012675.3, product 92 bp) and *Il6* (Assay Rn99999011_m1, GenBank NM_012589.2, product 90 bp). The reference genes were as follows: ribosomal protein S18 (*Rps18*, Assay Rn01428913_gH, GenBank MN_213557.1, product 62 bp), glyceraldehyde 3-phosphate dehydrogenase (*Gapdh*, Assay Rn1775763_g1, GenBank MN_017008.4, product 174 bp); and hypoxanthine-guanine phosphoribosyltransferase 1 (*Hprt1*, Assay Rn01527840_m1, GenBank MN_012583.2, product: 64 bp).

PCR was conducted in duplicate for each sample, and gene expression was determined by quantification relative to the reference gene. The calculation of the efficiencies for the target and control genes was performed using a relative standard curve with serial dilutions. The Pfaffl method was used (Pfaffl, 2001) for relative quantification of amplifications, the Pfaffl method was used (Pfaffl, 2001). The best combination of reference genes was selected using the NormFinder® program (MOMA, Denmark).

### Immunohistochemical analyses

Animal testis samples were used for immunostaining for TNFα and IL-6. In according with the characteristics of each antigen, antigen retrieval was performed by incubating the sections in citrate buffer using a pressure cooker for 10 minutes. To block endogenous peroxidase, the sections were subjected to a solution of hydrogen peroxide plus methanol (3% in methanol), and protein blocking was performed by incubation in a blocking solution with 5% bovine serum albumin (BSA) in TBS-T buffer at room temperature. Subsequently, the sections were subjected to reactions with specific primary antibodies against TNFα (1:50, 52B83, sc-257) and IL-6 (1:50, E-4, sc-28343) and incubated in a humid chamber overnight in a refrigerator at 4°C. The sections were incubated with secondary m-IgGK antibodies (1:200, IgG-HRP, sc-516 conjugate) at room temperature for 2 hours, incubated with diaminobenzidine (1:50 DAB diluted in 3% hydrogen peroxide for 3 minutes), counterstained with Harris hematoxylin for 3 minutes, and evaluated with a Zeiss Axiophoto photomicroscope (Zeiss, Munich, Germany). Images were acquired from 4 animals per group using a Zeiss Axiophoto photomicroscope (Zeiss, Munich, Germany) at 40× magnification. The intensity of TNFα and IL-6 antigen immunoreactivity was examined in 10 fields per animal using Image-J software version 1.50i (NIH, Bethesda, MD, USA), and the percentage of tissue marking was quantified for each image, and immunopositivity cells were used to calculate the area percentage (Jensen, 2013).

### Hormone dosages

After the animals were euthanized, blood (2 mL) was collected in tubes with EDTA anticoagulant, and the plasma was removed after centrifugation (2900 g/15 min) and stored in a freezer at -30°C. The plasma hormone levels were measured in duplicate. The radioimmunoassay for direct testosterone was conducted through a single assay using a kit (Immunotech - Beckman Couter, France), with a binding capacity of 60%, a sensitivity of 90% (0.02 ng/mL), and intra-assay coefficients of variation of 6.81% (low) and 4.49% (high). The corticosterone radioimmunoassay was performed through a single assay using a commercial kit specific for rats (MP Biomedicals Inc., USA), with a binding capacity of 57%, a sensitivity of 90% (1.52 ng/mL), and intra-assay coefficients of variation of 4.96% (low) and 3.35% (high). In contrast, prolactin was measured via a single enzyme immunoassay assay using a commercial rat kit (EZ International Trading Corp., USA), with a sensitivity of 89% (0.40 ng/mL) and intra-assay coefficients of variation of 4.68% (low) and 5.41% (high).

### Statistical analysis

The results were subjected to the Shapiro-Wilk test to verify the normality of the data. All variables were analyzed by the unpaired t-test, except for the testosterone concentration, which did not follow a normal distribution and was analyzed by the Mann-Whitney test. Differences were considered significant if P < 0.05.

## Results

**Figure 2:**
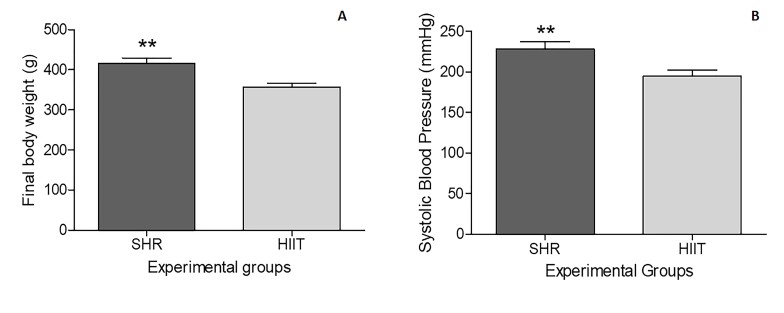
Means (± SEM) of the final body weight (g) and final systolic blood pressure (BSP) of SHRs subjected to HIIT on a treadmill. Body weight and BSP were assessed three days after the end of the HIIT program (five consecutive days each week over a period of 8 weeks; each session consisted of approximately 50 minutes). SHR (control group [n= 9]); and HIIT (trained group [n=10]). Unpaired t-test. ** Means with different subscripts differ (P < 0.01).

The animals in the HIIT group presented lower final body weights (P = 0.0017) and lower final BSPs (P = 0.0089) than did those in the SHR group, as shown in Fig. 2.

**Figure 3:**
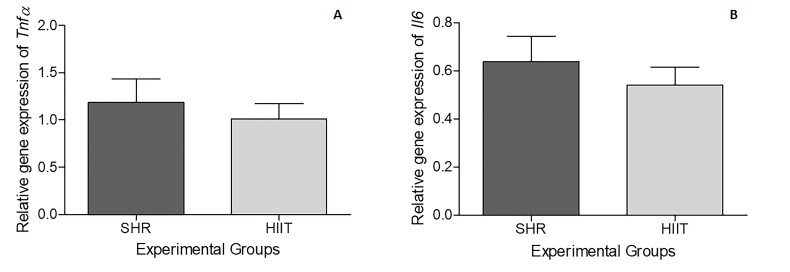
Mean (± SEM) fold change in gene expression (2-∆∆CT) in testicular tissue of SHRs subjected to HIIT on a treadmill. SHR (control group [n=9]) and HIIT (trained group [n=10]). Testes were assessed 2 days after the end of the HIIT program (five consecutive days each week over a period of 8 weeks; each session consisted of approximately 50 minutes). The combination of the reference genes Hprt1 and Rps18 was used as a normalization control for the reaction of the target genes *Tnfα* (A) and *Il6* (B). As revealed by an unpaired t test, no significant difference was found between the groups (P > 0.05).

For relative gene expression, the combination of *Hprt1* and *Rps18* was chosen as the reaction normalizer because of its stability in the NormFinder® program. However, none of the three reference genes tested (*Gapdh*, *Hprt1,* and *Rps18*) had values less than 0.5 in NormFinder® in this experimental design and could serve as reaction normalizers. No difference in the relative abundance of *Tnfα* (P = 0.5606) or *Il6* (P = 0.4617) mRNA in the testes was found between the two experimental groups (Fig. 3).

**Figure 4:**
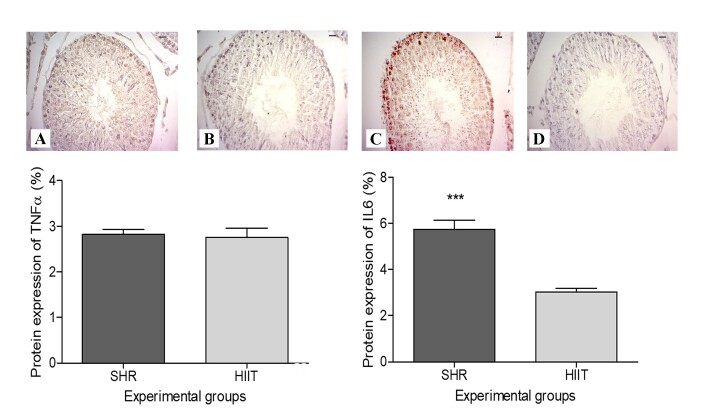
Protein expression of TNFα (A and B) and IL-6 (C and D) in the testes of SHRs subjected to HIIT on a treadmill. SHR (control group [n=9]) and HIIT (trained group [n=10]). Testes were assessed 2 days after the end of the HIIT program (five consecutive days each week over a period of 8 weeks; each session consisted of approximately 50 minutes). As determined by an unpaired t test no significant difference in the protein expression of TNFα was found between the groups (P = 0.8042). Asterisks indicate significant differences between the groups (***P = 0.0003).

According to the immunohistochemical analyses (Fig. 4), the testes of the animals in the SHR group showed greater IL-6 immunostaining (P = 0.0003) than did those of the animals in the HIIT group, although no difference in TNFα staining was found between the groups (P = 0.8042).

**Figure 5:**
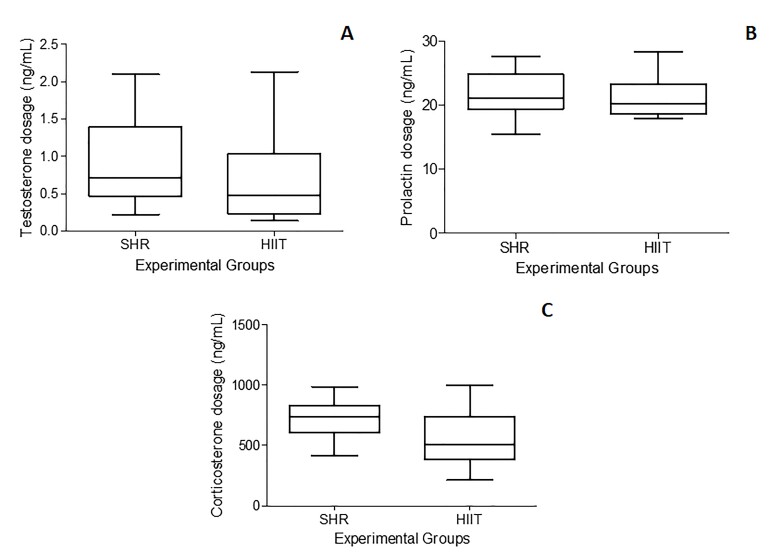
Plasma concentrations of testosterone, prolactin, and corticosterone in SHRs subjected to HIIT on a treadmill. SHR (control group [n=9]) and HIIT (trained group [n=10]). Testes were assessed 2 days after the end of the HIIT program (five consecutive days each week over a period of 8 weeks; each session consisted of approximately 50 minutes). An unpaired t test was used for the analysis of corticosterone and prolactin, and the Mann-Whitney test was used for the analysis of testosterone. No difference was found between the groups (P > 0.05).

The plasma concentrations of testosterone, prolactin, and corticosterone did not differ between the two experimental groups (SHR and HIIT; P = 0.4622, P = 0.7643, and P = 0.0872, respectively). The data are shown in Fig. 5.

## Discussion

Hypertension was confirmed in our experiment, as all the rats had BSP values above 140 mmHg before and after the experimental period, which is characteristic of hypertension in rats (Kapsdorferová et al., 2024). As expected, HIIT decreased body weight and SBP in hypertensive animals. HIIT effectively reduces body weight and SBP (Engel et al., 2022; Oliveira et al., 2023). SHRs are born normotensive but start to develop hypertension at 2 to 4 months of life and already have sustained hypertension at 6 months of age (Azu, 2015); at the beginning of this experiment, the animals were 12 months of age. In addition to high blood pressure, SHRs have multiple microvascular complications that alter vascular structure and function and can affect animal fertility (Azu, 2015).

Although the gene expression data revealed a decreasing trend in the abundance of *Il6* mRNA in the HIIT group, no significant difference was detected between the groups. However, a significant reduction in the synthesis of IL6 was observed in the testes of rats with hypertension subjected to HIIT, demonstrating that this modality of physical training can effectively reduce the levels of testicular inflammatory mediators caused by hypertension. Hypertension causes gonadal macrophage imbalance (M1 increases and M2 decreases), inflammation, lymphangiogenesis and reproductive dysfunction (Navaneethabalakrishnan et al., 2022). In a model of chronic testicular inflammation (experimental autoimmune orchitis) an increase in M1 macrophages in the testes and increased levels of monocyte chemoattractant protein-1 (MCP-1), IL-6 and TNFα (Nicolas et al., 2017) were observed. Hypertensive mice present a lower expression pattern of several steroidogenic enzymes and hormone receptors, higher expression pattern of proinflammatory mediators IL-1b, IL-6, IL-17, TNFα and nitric oxide synthase (NOS2) in testes and sperm alterations (Navaneethabalakrishnan et al., 2022).

Studies have shown that the IL-6 level is increased in the seminal plasma of men with impaired fertility (Djourabchi Borojerdi et al., 2020). Moreover, there are increased levels of ROS in the semen of infertile male patients (Iwasaki and Gagnon, 1992). Cytokines act together in a network that can impair spermatogenesis (Seshadri et al., 2009). A negative correlation has been found for sperm motility with IL-6 in seminal plasma (Kopa et al., 2005), and with IL-6 receptors on spermatozoids (Djourabchi Borojerdi et al., 2020). In addition, a decrease in sperm motility has been observed with elevated IL-6 (Djourabchi Borojerdi et al., 2020). IL-6 can be produced by Sertoli cells, Leydig cells, germ cells, peritubular myiod cells, prostate cells and interstitial macrophages (Camejo et al., 2001; Hakovirta et al., 1995; Wang et al., 2018).

During sperm production, cytoplasmic fragments (lipopolysaccharide and residual bodies) of spermatids are released and stimulate Sertoli cells to produce IL-1α, which induces Sertoli cells to secrete IL-6 (Syed et al., 1995). However, in contrast to IL-1α, IL-6 may be a potent inhibitor of premeiotic DNA synthesis within the seminiferous epithelium (Hakovirta et al., 1995). IL-6 is involved in germ cell apoptosis in testis inflammation (Rival et al., 2006) and exogenous IL-6 can inhibit testosterone production and stem Leydig cell development (Wang et al., 2018).

In the present study, HIIT did not alter the expression of TNFα in the testes of hypertensive rats. TNFα is produced by macrophages, endothelial cells, Sertoli cells, and germ cells (Camejo et al., 2001; Li et al., 2006), and its receptors are located in Sertoli cells (Li et al., 2006). This cytokine regulates different cellular processes pertinent to spermatogenesis, although TNFα induces germ cell loss from seminiferous epithelium and causes transient disruption of blood-testis barrier integrity (Li et al., 2006).

HIIT did not alter the plasma concentrations of testosterone, prolactin or corticosterone in SHRs. Physical exercise promotes adaptations of the endocrine system, mainly regarding the secretion of testosterone and cortisol (Hackney and Lane, 2015). The effects of exercise on testosterone and cortisol levels vary across exercise modalities and can be affected by exercise intensity and volume, nutritional intake, and training experience (Kraemer and Ratamess, 2005). Protocols of sufficient intensity and volume produce substantial elevations in total testosterone; however, testosterone did not significantly increase until after the fourth set was completed (Bosco et al., 2000). In humans, a recent meta-analysis demonstrated that testosterone and cortisol increase immediately after a single HIIT session, then decrease below baseline levels, and finally return to baseline values after 24 h (Dote‐Montero et al., 2021).

SHRs have blood testosterone levels equivalent to those of normotensive Wistar-Kyoto rats (strain of origin of the SHR) (Kozłowska et al., 2019) and greater than those found in normotensive Long-Evans rats (Clark et al., 1991). Hypertension is correlated with low testosterone levels in men (Erenpreiss et al., 2020; Kelly and Jones, 2013; Wang et al., 2011); however, men have a greater incidence of hypertension and higher blood pressure than premenopausal women do (Sandberg and Ji, 2012). Similarly, young female SHRs have a lower BSP than male SHRs do, and once female SHRs stop cycling (10-12 months), BP increases until the sex difference in BP is abolished (16-18 months) (Elmarakby and Sullivan, 2021). There is a relationship between hormones and blood pressure, but the mechanisms underlying sex differences in BSP remain unclear (Elmarakby and Sullivan, 2021).

The chromosomal locus responsible for the high blood pressure of SHRs is located on the Y chromosome, and its maximum expression depends on testosterone and the androgen receptor (Johnson et al., 1995). The offspring of SHRs mated with Wistar-Kyoto rats have greater blood pressure when the father is an SHR (Ely and Turner, 1990). The development of hypertension is attenuated by castration or testosterone receptor antagonism in young male SHRs (Ganten et al., 1989). Testosterone reduces the ability of the kidney to produce a natriuretic response in both male and female SHR (Reckelhoff et al., 1998). The etiology of infertility in SHRs is likely related to vascular damage (Azu, 2015).

The steroid sulfatase enzyme (STS) is responsible for the hydrolysis of the sulfate of steroids to transform the sulfates of steroid sex hormones to free active steroids (Johnson et al., 1995). Circulating dehydroepiandrosterones can be transported into the cell, desulfated by STS and then metabolized into testosterone and dihydrotestosterone (Mueller et al., 2015). The activity of STS is increased in the testes of SHRs (Johnson et al., 1995).

We did not observed alteration in prolactin or corticosterone concentrations in trained SHR. The levels of both of these hormones increase under acute stress situations (Wexler et al., 1980). Physical exercise stress increases plasma concentrations of corticosterone and prolactin, and there is evidence that these hormones stimulate the phagocytic activity of macrophages, but this phenomenon is more evident in high-intensity exercise without previous training (Ortega et al., 1996).

Tan and Hutchinson (Tan and Hutchinson, 1987) reported no significant differences in prolactin levels between SHRs and Wistar-Kyoto rats, although SHRs had higher concentrations of prolactin. SHRs also respond to stress by increasing their levels of prolactin (Wexler et al., 1980). Prolactin increases the responsiveness of Leydig cells to luteinizing hormone (LH), and LH treatment combined with prolactin increases testosterone production in rats (Bartke and Dalterio, 1976).

SHRs have higher concentrations of corticosterone and cortisol than 10-week-old Wistar-Kyoto rats do (Kozłowska et al., 2019), probably because glucocorticoids are involved in blood pressure regulation (Ohanian and Heagerty, 1992). Cortisol and corticosterone are related to the inflammatory response, and an increase in their levels leads to decreases in the levels of inflammatory cytokines (Kozłowska et al., 2019). However, no increase in plasma corticosterone was observed in the group that underwent HIIT, and significant decreases in the levels of inflammatory cytokines were detected.

## Conclusion

HIIT is beneficial for decreasing SBP and IL-6 levels in the testes of hypertensive individuals without changing the plasma concentrations of testosterone, prolactin, or corticosterone.

## Data Availability

Research data is only available upon request.

## References

[B001] Akomolafe SF, Olasehinde TA, Oladapo IF, Oyeleye SI (2022). Diet supplemented with Chrysophyllum albidum G. Don (Sapotaceae) fruit pulp improves reproductive function in hypertensive male rats. Reprod Sci.

[B002] Altıntaş Aykan D (2020). The effects of sacubitril/valsartan and ramipril on the male fertility in hypertensive rats. North Clin Istanb.

[B003] Azu OO (2015). Testicular morphology in spontaneously hypertensive rat model: oxidant status and stereological implications. Andrologia.

[B004] Bartke A, Dalterio S (1976). Effects of prolactin on the sensitivity of the testis to LH1. Biol Reprod.

[B005] Bosco C, Colli R, Bonomi R, Von Duvillard SP, Viru A (2000). Monitoring strength training: neuromuscular and hormonal profile. Med Sci Sports Exerc.

[B006] Camejo MI, Segnini A, Proverbio F (2001). Interleukin-6 (il-6) in seminal plasma of infertile men, and lipid peroxidation of their sperm. Arch Androl.

[B007] Ciolac EG, Bocchi EA, Greve JMD, Guimaraes GV (2011). Heart rate response to exercise and cardiorespiratory fitness of young women at high familial risk for hypertension: effects of interval vs continuous training. Eur J Cardiovasc Prev Rehabil.

[B008] Clark JT, Sahu A, Mrotek JJ, Kalra SP (1991). Sexual function and neuropeptide Y levels in selected brain regions in male spontaneously hypertensive rats. Am J Physiol.

[B009] Colli LG, Belardin LB, Echem C, Akamine EH, Antoniassi MP, Andretta RR, Mathias LS, Rodrigues SFP, Bertolla RP, de Carvalho MHC (2019). Systemic arterial hypertension leads to decreased semen quality and alterations in the testicular microcirculation in rats. Sci Rep.

[B010] Correia RR, Batista VRG, Veras ASC, Tavares MEA, Souza FLA, Pacagnelli FL, Campos DHS, Giometti IC, Teixeira GR (2022). High‐intensity interval training attenuates the effects caused by arterial hypertension in the ventral prostate. Prostate.

[B011] Didion SP (2017). Cellular and oxidative mechanisms associated with interleukin-6 signaling in the vasculature. Int J Mol Sci.

[B012] Djourabchi Borojerdi AS, Welchowski T, Peng W, Buchen A, Novak N, Haidl G, Duan Y, Allam J (2020). Human spermatozoa of male patients with subfertility express the interleukin‐6 receptor. Andrologia.

[B013] Dote‐Montero M, Carneiro‐Barrera A, Martinez‐Vizcaino V, Ruiz JR, Amaro‐Gahete FJ (2021). Acute effect of HIIT on testosterone and cortisol levels in healthy individuals: a systematic review and meta‐analysis. Scand J Med Sci Sports.

[B014] Elmarakby AA, Sullivan JC (2021). Sex differences in hypertension: lessons from spontaneously hypertensive rats (SHR). Clin Sci (Lond).

[B015] Ely DL, Turner ME (1990). Hypertension in the spontaneously hypertensive rat is linked to the Y chromosome. Hypertension.

[B016] Engel LE, de Souza FLA, Giometti IC, Okoshi K, Mariano TB, Ferreira NZ, Pinheiro DG, Floriano RS, Aguiar AF, Cicogna AC, Vechetti IJ, Pacagnelli FL (2022). The high-intensity interval training mitigates the cardiac remodeling in spontaneously hypertensive rats. Life Sci.

[B017] Erenpreiss J, Fodina V, Pozarska R, Zubkova K, Dudorova A, Pozarskis A (2020). Prevalence of testosterone deficiency among aging men with and without morbidities. Aging Male.

[B018] Ferreira NZ, Agostinho de Souza FL, Mariano TB, Carrara B, Collegio G, Molinari AO, Martins C, Dias R, Moschini G, Thomaz GEN, Pinheiro DG, Giometti IC, Pacagnelli FL (2017). Efeito hipotensor do exercício intervalado de alta intensidade em animais espontaneamente hipertensos. Colloq Vitae.

[B019] Foy CG, Newman JC, Berlowitz DR, Russell LP, Kimmel PL, Wadley VG, Thomas HN, Lerner AJ, Riley WT (2019). Blood pressure, sexual activity, and erectile function in hypertensive men: baseline findings from the Systolic Blood Pressure Intervention Trial (SPRINT). J Sex Med.

[B020] Frieden TR, Jaffe MG (2018). Saving 100 million lives by improving global treatment of hypertension and reducing cardiovascular disease risk factors. J Clin Hypertens (Greenwich).

[B021] Ganten U, Schröder G, Witt M, Zimmermann F, Ganten D, Stock G (1989). Sexual dimorphism of blood pressure in spontaneously hypertensive rats. J Hypertens.

[B022] Guide for the Care and Use of Laboratory Animals (2011). National Research Council (US) Committee for the Update of the Guide for the Care and Use of Laboratory Animals..

[B023] Guo D, Li S, Behr B, Eisenberg ML (2017). Hypertension and male fertility. World J Mens Health.

[B024] Guzik TJ, Touyz RM (2017). Oxidative stress, inflammation, and vascular aging in hypertension. Hypertension.

[B025] Hackney AC, Lane AR (2015). Exercise and the regulation of endocrine hormones. Prog Mol Biol Transl Sci.

[B026] Hakovirta H, Syed V, Jégou B, Parvinen M (1995). Function of interleukin-6 as an inhibitor of meiotic DNA synthesis in the rat seminiferous epithelium. Mol Cell Endocrinol.

[B027] Han R-Y, Ma J, Ma J-Y, Wang X-C, An X-T, Zhang Z-D, Wang S-S (2017). Correlation of semen parameters with inflammatory factors in the seminal plasma of obese males. J Androl.

[B028] Handy DE, Loscalzo J (2017). Responses to reductive stress in the cardiovascular system. Free Radic Biol Med.

[B029] Hussein MR, Abou-Deif ES, Bedaiwy MA, Said TM, Mustafa MG, Nada E, Ezat A, Agarwal A (2005). Phenotypic characterization of the immune and mast cell infiltrates in the human testis shows normal and abnormal spermatogenesis. Fertil Steril.

[B030] Iwasaki A, Gagnon C (1992). Formation of reactive oxygen species in spermatozoa of infertile patients. Fertil Steril.

[B031] Jensen EC (2013). Quantitative analysis of histological staining and fluorescence using imageJ. Anat Rec (Hoboken).

[B032] Johnson ML, Ely DL, Turner ME (1995). Steroid sulfatase and the Y chromosome hypertensive locus of the spontaneously hypertensive rat. Steroids.

[B033] Kapsdorferová V, Grešová S, Švorc P (2024). Measurement of blood pressure in rats: invasive or noninvasive methods?. Physiol Rep.

[B034] Kelly DM, Jones TH (2013). Testosterone: a metabolic hormone in health and disease. J Endocrinol.

[B035] Kopa Z, Wenzel J, Papp GK, Haidl G (2005). Role of granulocyte elastase and interleukin-6 in the diagnosis of male genital tract inflammation. Andrologia.

[B036] Kozłowska A, Wojtacha P, Równiak M, Kolenkiewicz M, Tsai M-L (2019). Differences in serum steroid hormones concentrations in Spontaneously Hypertensive Rats (SHR) – an animal model of Attention-Deficit/Hyperactivity Disorder (ADHD). Physiol Res.

[B037] Kraemer WJ, Ratamess NA (2005). Hormonal responses and adaptations to resistance exercise and training. Sports Med.

[B038] Li MWM, Xia W, Mruk DD, Wang CQF, Yan HHN, Siu MKY, Lui W, Lee WM, Cheng CY (2006). Tumor necrosis factor α reversibly disrupts the blood–testis barrier and impairs Sertoli–germ cell adhesion in the seminiferous epithelium of adult rat testes. J Endocrinol.

[B039] Li W, Wang D, Wu C, Shi O, Zhou Y, Lu Z (2017). The effect of body mass index and physical activity on hypertension among Chinese middle-aged and older population. Sci Rep.

[B040] MacDonald C, Madika A, Lajous M, Laouali N, Artaud F, Bonnet F, Fagherazzi G, Boutron‐Ruault M (2020). Associations between physical activity and incident hypertension across strata of body mass index: a prospective investigation in a large cohort of french women. J Am Heart Assoc.

[B041] Micheli L, Collodel G, Cerretani D, Menchiari A, Noto D, Signorini C, Moretti E (2019). Relationships between ghrelin and obestatin with MDA, proinflammatory cytokines, GSH/GSSG ratio, catalase activity, and semen parameters in infertile patients with leukocytospermia and varicocele. Oxid Med Cell Longev.

[B042] Mills KT, Bundy JD, Kelly TN, Reed JE, Kearney PM, Reynolds K, Chen J, He J (2016). Global disparities of hypertension prevalence and control: a systematic analysis of population-based studies from 90 countries. Circulation.

[B043] Moreira JBN, Bechara LRG, Bozi LHM, Jannig PR, Monteiro AWA, Dourado PM, Wisløff U, Brum PC (2013). High- versus moderate-intensity aerobic exercise training effects on skeletal muscle of infarcted rats. J Appl Physiol.

[B044] Mueller JW, Gilligan LC, Idkowiak J, Arlt W, Foster PA (2015). The regulation of steroid action by sulfation and desulfation. Endocr Rev.

[B045] Navaneethabalakrishnan S, Goodlett BL, Lopez AH, Rutkowski JM, Mitchell BM (2020). Hypertension and reproductive dysfunction: a possible role of inflammation and inflammation-associated lymphangiogenesis in gonads. Clin Sci (Lond).

[B046] Navaneethabalakrishnan S, Wilcox BK, Goodlett BL, Murphy MM, Mitchell BM (2022). Hypertension induces gonadal macrophage imbalance, inflammation, lymphangiogenesis, and dysfunction. Clin Sci (Lond).

[B047] Nicolas N, Michel V, Bhushan S, Wahle E, Hayward S, Ludlow H, de Kretser DM, Loveland KL, Schuppe H-C, Meinhardt A, Hedger MP, Fijak M (2017). Testicular activin and follistatin levels are elevated during the course of experimental autoimmune epididymo–orchitis in mice. Sci Rep.

[B048] Ohanian J, Heagerty AM (1992). The phosphoinositide signaling system and hypertension. Curr Opin Nephrol Hypertens.

[B049] Oja P, Kelly P, Pedisic Z, Titze S, Bauman A, Foster C, Hamer M, Hillsdon M, Stamatakis E (2017). Associations of specific types of sports and exercise with all-cause and cardiovascular-disease mortality: a cohort study of 80 306 British adults. Br J Sports Med.

[B050] Oliveira GH, Okawa RTP, Simões CF, Locatelli JC, Mendes VHS, Reck HB, Lopes WA (2023). Efeitos do treinamento intervalado de alta intensidade sobre a pressão arterial central: uma revisão sistemática e metanálise. Arq Bras Cardiol.

[B051] Ortega E, Rodríguez M, Barriga C, Forner M (1996). Corticosterone, prolactin and thyroid hormones as hormonal mediators of the stimulated phagocytic capacity of peritoneal macrophages after high-intensity exercise. Int J Sports Med.

[B052] Pacagnelli FL, Okoshi K, Campos DHS, Alves De Souza RW, Padovani CR, Carvalho RF, Aguiar AF, Dal Pai M, Cicogna AC (2014). Physical training attenuates cardiac remodeling in rats with supra-aortic stenosis. Exp Clin Cardiol.

[B053] Pfaffl MW (2001). A new mathematical model for relative quantification in real-time RT-PCR. Nucleic Acids Res.

[B054] Reckelhoff JF, Zhang H, Granger JP (1998). Testosterone exacerbates hypertension and reduces pressure-natriuresis in male spontaneously hypertensive rats. Hypertension.

[B055] Rival C, Theas MS, Guazzone VA, Lustig L (2006). Interleukin-6 and IL-6 receptor cell expression in testis of rats with autoimmune orchitis. J Reprod Immunol.

[B056] Rossoni LV, Oliveira RAF, Caffaro RR, Miana M, Sanz-Rosa D, Koike MK, Do Amaral SL, Michelini LC, Lahera V, Cachofeiro V (2011). Cardiac benefits of exercise training in aging spontaneously hypertensive rats. J Hypertens.

[B057] Saco‐Ledo G, Valenzuela PL, Ruiz‐Hurtado G, Ruilope LM, Lucia A (2020). Exercise reduces ambulatory blood pressure in patients with hypertension: a systematic review and meta‐analysis of randomized controlled trials. J Am Heart Assoc.

[B058] Sandberg K, Ji H (2012). Sex differences in primary hypertension. Biol Sex Differ.

[B059] Seshadri S, Bates M, Vince G, Jones DIL (2009). The role of cytokine expression in different subgroups of subfertile men. Am J Reprod Immunol.

[B060] Sun Z (2015). Aging, arterial stiffness, and hypertension. Hypertension.

[B061] Syed V, Stéphan JP, Gérard N, Legrand A, Parvinen M, Bardin CW, Jégou B (1995). Residual bodies activate Sertoli cell interleukin-1 alpha (IL-1 alpha) release, which triggers IL-6 production by an autocrine mechanism, through the lipoxygenase pathway. Endocrinology.

[B062] Tan BKH, Hutchinson JS (1987). Plasma and pituitary prolactin and blood pressure in bromocriptine-treated spontaneously hypertensive and Wistar-Kyoto rats. Clin Exp Pharmacol Physiol.

[B063] Tjønna AE, Lee SJ, Rognmo Ø, Stølen TO, Bye A, Haram PM, Loennechen JP, Al-Share QY, Skogvoll E, Slørdahl SA, Kemi OJ, Najjar SM, Wisløff U (2008). Aerobic interval training versus continuous moderate exercise as a treatment for the metabolic syndrome: A pilot study. Circulation.

[B064] Wang C, Jackson G, Jones TH, Matsumoto AM, Nehra A, Perelman MA, Swerdloff RS, Traish A, Zitzmann M, Cunningham G (2011). Low testosterone associated with obesity and the metabolic syndrome contributes to sexual dysfunction and cardiovascular disease risk in men with type 2 Diabetes. Diabetes Care.

[B065] Wang Y, Chen L, Xie L, Li L, Li X, Li H, Liu J, Chen X, Mao B, Song T, Lian Q, Ge R-S (2018). Interleukin 6 inhibits the differentiation of rat stem Leydig cells. Mol Cell Endocrinol.

[B066] Wen CP, Wai JPM, Tsai MK, Yang YC, Cheng TYD, Lee M-C, Chan HT, Tsao CK, Tsai SP, Wu X (2011). Minimum amount of physical activity for reduced mortality and extended life expectancy: a prospective cohort study. Lancet.

[B067] Wexler BC, Iams SG, McMurtry JP (1980). Pathophysiological differences between obese and non-obese spontaneously hypertensive rats. Br J Exp Pathol.

[B068] Yi X, Tang D, Cao S, Li T, Gao H, Ma T, Yao T, Li J, Chang B (2020). Effect of different exercise loads on testicular oxidative stress and reproductive function in obese male mice. Oxid Med Cell Longev.

